# 258. D-Dimer, Erythrocyte Sedimentation Rate, and C-Reactive Protein Sensitivity for PJI Diagnosis

**DOI:** 10.1093/ofid/ofab466.460

**Published:** 2021-12-04

**Authors:** Ayden Case, Lefko T Charalambous, Trevor Bowman, Ian Duensing, Edward Hendershot, Jessica Seidelman, Thorsten Seyler, William Jiranek

**Affiliations:** 1 Duke University, Durham, North Carolina; 2 Duke University Medical Center, Dept. of Orthopaedics, Durham, North Carolina; 3 duke university, Durham, North Carolina

## Abstract

**Background:**

Consensus criteria for the diagnosis of acute PJI now include D-dimer. Additionally, Erythrocyte Sedimentation Rate (ESR) is of questionable use in the diagnosis of acute PJI. There is scarce and contradicting evidence on the diagnostic value for these biomarkers, and further studies on larger cohorts are needed for validation. We sought to quantify the sensitivities of D-dimer and ESR compared to C-Reactive Protein (CRP) in the diagnosis of acute PJI at a tertiary referral center.

Sensitivity Table for D-Dimer and ESR

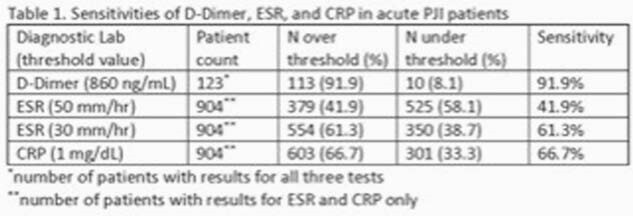

**Methods:**

An institutional database was queried for patients undergoing revision procedures for PJI after total hip arthroplasty (THA) and total knee arthroplasty (TKA) from 2014 to present. Patients were included if they had a PJI diagnosis code with subsequent revision procedure CPT codes and PICC line placement within 21 days of revision surgery. Patients with inflammatory arthropathies were excluded. Diagnostic labs, including CRP, ESR, and D-dimer, were collected within 90 days pre- and post-operatively and sensitivities for the diagnosis of PJI were calculated. Cutoff values included CRP >1 mg/dL, ESR >30 mm/hr and >50 mm/hr, and D-dimer >860 ng/mL.

**Results:**

In total, 961 PJI patients were identified. Of those, 904 had ESR and CRP values collected, and 123 had ESR, CRP, and D-dimer collected. In the cohort of patients with ESR and CRP, 603 patients had elevated CRP, 554 had ESR >30 mm/hr, and 379 had ESR >50 mm/hr, corresponding to sensitivities of 66.7%, 61.3%, and 41.9%, respectively. In the cohort of patients with all three biomarkers, 113 had an elevated D-dimer, corresponding to a sensitivity of 91.9%.

**Conclusion:**

In this cohort, CRP and ESR were of comparable sensitivity in diagnosing PJI. D-dimer was the most sensitive, but further pooled studies are needed to confirm this. Providers should continue to use this information in the context of other data and MSIS criteria to inform decision-making.

**Disclosures:**

**Thorsten Seyler, MD/PhD**, **Depuy Synthes** (Other Financial or Material Support, Resident Educational Support)**Extrel Therapeutics** (Board Member, Shareholder)**Heraeus Medical** (Consultant)**MiCare Path** (Board Member, Shareholder)**OREF** (Grant/Research Support)**Pattern health** (Board Member)**Restor3D** (Other Financial or Material Support, Royalties)**Smith+Nephew, Inc.** (Grant/Research Support, Speaker’s Bureau)**Stryker** (Other Financial or Material Support, Resident Educational Support)**Total Joint Orthopedics, Inc.** (Consultant)**Wolters Kluwer Health** (Other Financial or Material Support, Royalties)**Zimmer Biomet** (Grant/Research Support) **William Jiranek, MD**, **Depuy Synthes** (Other Financial or Material Support, Royalty/Licensing)

